# Higher temporal evapotranspiration estimation with improved SEBS model from geostationary meteorological satellite data

**DOI:** 10.1038/s41598-019-50724-w

**Published:** 2019-10-18

**Authors:** Jing Zhao, Xuelong Chen, Jing Zhang, Honggang Zhao, Yongyu Song

**Affiliations:** 1 Beijing Key Laboratory of Resource Environment and Geographic Information System, Capital Normal University, Beijing, 100048 China; 2 Faculty of Geo-Information Science and Earth Observation, University of Twente, Enschede, 7514AE The Netherlands; 3 Institute of Tibetan Plateau Research, Chinese Academy of Sciences, Beijing, 100101 China; 4 Xinjiang Normal University, Xinyi Road, Urumqi, 830046 China

**Keywords:** Climate sciences, Hydrology

## Abstract

Evapotranspiration (ET) is a key variable in hydrologic cycle that directly affects the redistribution of precipitation and surface balance. ET measurements with high temporal resolution are required for coupling with models of highly dynamic processes, e.g., hydrological and land surface processes. The Haihe River Basin is the focus of China’s industrial base and it is one of the three major grain-producing regions within the country. However, this area is facing serious water resource shortages and water pollution problems. The present study used geostationary satellite remote sensing data, *in situ* meteorological observations, and the surface energy balance system (SEBS) model with a new kB^−1^ parameterization to estimate 3-hourly and daily energy and water fluxes in the Haihe River Basin. The results of the SEBS model were validated with point-scale data from five observation flux towers. Validation showed that 3-hourly and daily ET derived from the SEBS model performed well (R^2^ = 0.67, mean bias = 0.027 mm/h, RMSE = 0.1 mm/h). Moreover, factors influencing ET were also identified based on the results of this study. ET varies with land cover type and physical and chemical properties of the underlying surface. Furthermore, ET is also controlled by water availability, radiation, and other atmospheric conditions. It was found that ET had strong correlation with the normalized difference vegetation index (NDVI). Specifically, daily ET fluctuated with the NDVI when the NDVI was <0.29, and ET increased rapidly as the NDVI increased from 0.29 to 0.81. For NDVI values >0.81, indicating a state of saturation, the rate of increase of ET slowed. This research produced reliable information that could assist in sustainable management of the water resources and in improved understanding of the hydrologic cycle of the Haihe River Basin.

## Introduction

Water is a critical factor in restricting both economic development and ecological restoration, and it constitutes a core component of environmental and ecosystem research^1–4^. Evapotranspiration (ET) is one of the most important part of the water cycle, especially in semiarid inland areas, and it has a crucial role in measures adopted to save agricultural water, manage water resources, and monitor drought^5–8^. ET is a central process in the climate system and it represents a nexus of the water, energy, and carbon cycles^9^. Therefore, ET is of considerable importance in terms of understanding climate change and in comprehending the associated effects on water resource management. Ground-based observation systems typically use monitoring instruments that are reasonably expensive and can only observe ET over homogenous surfaces within a few kilometres of a point of interest, which clearly limits our ability to map ET over heterogeneous surfaces.

Estimations of the spatial distribution of ET are essential components of general circulation and hydrologic models^10,11^. Measurements of ET can be obtained from either ground-based instruments or remote sensing (RS) satellite observations. Typically, ground-based observational techniques (e.g., lysimeters, the Bowen ratio energy balance method, and eddy covariance (EC) systems) require advanced instrumentation, as well as substantial data screening and interpretation^12,13^.

In recent decades, advancements in RS technologies have provided new possibilities for the acquisition of spatially distributed regional ET information^14^. Numerous attempts have been made to develop approaches and models to extract ET information from RS imagery^15–18^. Methods that have been developed include the surface energy balance algorithm for land^19^, two-source energy balance^20,21^, surface energy balance system (SEBS) model^22^, and satellite-based energy balance for mapping ET with internalized calibration^23^. In these models, land surface parameters play a crucial role in obtaining reliable estimations of ET. These models require explicit characterization of numerous physical parameters, many of which are difficult to determine. In comparison with low-resolution satellite imagery, the use of images with high temporal and spatial resolutions can reduce the uncertainties of model input parameters. Many RS-based ET calculations use Moderate Resolution Imaging Spectroradiometer (MODIS) and high-spatial-resolution data from satellites such as Landsat and Advanced Spaceborne Thermal Emission and Reflection Radiometer (ASTER)^24,25^. However, the primary disadvantage of using such data in diagnostic modelling is that they often suffer large spatial and temporal gaps because of cloud cover and infrequent image availability determined by the satellite overpass schedule. Geostationary satellites have high temporal resolution and they can provide hourly and daily land surface information^26,27^, offering strong potential for the calculation of land surface and water cycle data at hourly temporal resolution. Therefore, it would be advantageous to use geostationary satellite data as model input parameters for calculation of ET with improved temporal resolution.

High-temporal-scale data are required for modelling highly dynamic processes, e.g., hydrological and land surface processes. Therefore, further effort is needed to advance the estimation of ET with high temporal resolution. Generally, a geostationary sensor can provide time-consistent information on hourly and daily timescales scales because sampling is typically performed at 30-min intervals. Although the frequency of image acquisition by sensors onboard geostationary satellites can capture the diurnal variations of land surface variables, the use of geostationary satellite data for ET research remains a challenge. Recent studies have highlighted the potential of using geostationary satellite data for such purposes, and there is growing interest in RS retrievals of ET using geostationary satellite data^28,29^. It is possible to use such data together with ground-based meteorological observations to calculate high-temporal-resolution (i.e., hourly) ET for a specific study area for water resources management and better understanding of land surface hydrological process. The present study focused on the use of data from the geostationary Multifunctional Transport Satellite (MTSAT), in combination with ground-based meteorological observations, for the estimation of hourly ET. In addition, comparison was made between the SEBS/RS-derived ET and the ground-observed ET.

The Haihe River Basin is China’s most important industrial base and it is one of the three major grain-producing regions of the country. However, this area has serious problems in terms of water resource shortages, water pollution, and ecological environment deterioration^30,31^. Furthermore, estimation of ET is difficult in this area because of the complicated underlying surface conditions. Therefore, there is urgent need for scientific and quantitative investigation to provide enhanced understanding of the components of the basin’s water balance and of their spatial distribution and temporal change. However, most previous studies on ET in the region have focused either on annual or monthly time or on specific dates^32–34^. Few studies have considered high-temporal-resolution (hourly) ET data that could be coupled effectively with hydrological and land surface process models of this area.

The specific objectives of the present study were as follows: 1) quantification of hourly and daily ET in the Haihe River Basin using the SEBS model in combination with the new KB^−1^ (a parameter used to calculate sensible heat flux) parameterization methods and geostationary thermal RS data; 2) comparison of estimates of ET with *in situ* measurements; and 3) investigation of the role of model input parameters/variables derived from geostationary satellite RS data in ET accuracy. The findings of this study also further improve on recent research achievements in hydrology and meteorology, promote the rational use of water, and provide basic data for the management of water resources in the Haihe River Basin.

## Results

### Validation with energy flux results

To analyse the reliability of the energy flux results, we validated the estimates of net radiation (R_n_), ground heat flux (G_0_), sensible heat flux (H), latent heat flux (LE), and hourly ET against point measurements from all observation stations. To minimize errors, this study considered all cloud-free days during 2014–2016. The validation of R_n_, G_0_, H, and LE used instantaneous values. The mean statistical error analysis for all stations (Table 1) shows that R_n_ has larger values of R^2^ (0.94) and mean bias MB (−16.35 W/m^2^) than G_0_, H, and LE. The validation results for Daxing station are much better than for the other stations. The performance in estimating R_n_ is satisfactory at all stations. For LE, the R^2^ value of the validation result is 0.83 at Miyun station with an MB of 6.63 W/m^2^; this result is better compared to the other stations.

**Table 1:** Statistical results of error analysis on 3-hourly SEBS estimates from five flux towers.

	R_n_ (W/m^2^)	G_0_ (W/m^2^)	H (W/m^2^)	LE (W/m^2^)	ET(mm/3 h)
Slope	1.0	0.9	0.9	1.1	1.4
Intercept	−13.25	10.28	0.57	9.48	0.03
RMSE	50.73	33.83	47.06	57.93	0.32
R^2^	0.94	0.63	0.58	0.65	0.67
MB	−16.35	10.57	−3.32	11.72	0.08
Sample	2615	1759	1442	1930	1490

*RMSE: root mean squared error, MB: mean bias.

**Figure 1:**
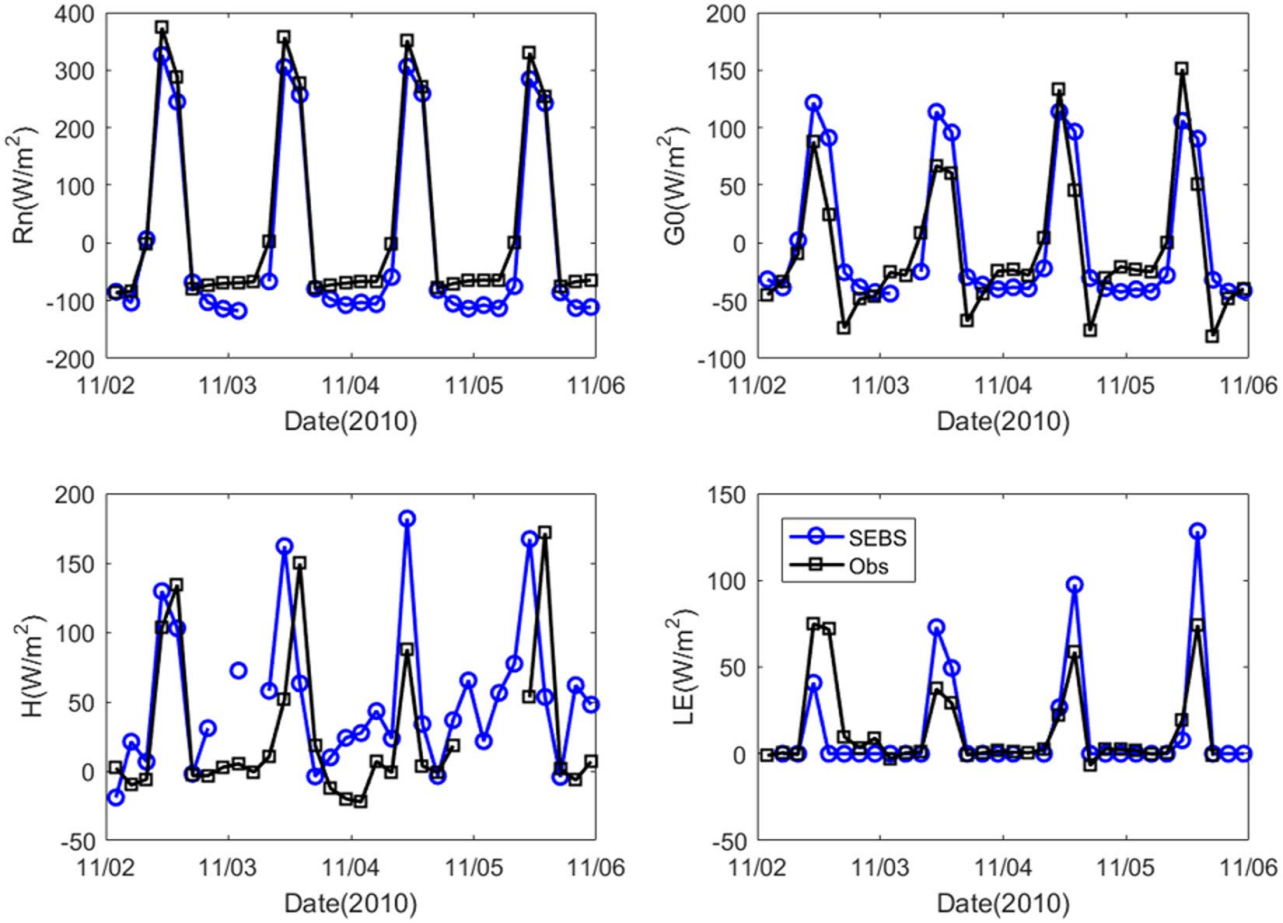
Time series comparison of SEBS output against measurements (Obs) at Miyun station: (**a**) net radiation (R_n_), (**b**) soil heat flux (G_0_,), (**c**) sensible heat flux (H), and (**d**) latent heat flux (LE).

Figure 1 indicates that the trend of the energy fluxes (R_n_, G_0_, H, and LE) from the SEBS model is similar to the trend of the observations. The driving force of ground energy, material transport, and exchange processes is R_n_ and therefore its variation alters other components of the surface heat balance. It is calculated mainly from the incident and reflected total solar shortwave radiation and the ground longwave radiation. The estimate of R_n_ from the SEBS model is lower than the station observations because of atmospheric effects. In contrast, the SEBS estimate of H is greater than the station observations. The range of H is large, which reflects the complexity of the underlying surface and the heterogeneous distribution of land surface temperature and wind speed. Therefore, it remains a challenge to produce high-temporal-resolution estimates of H for the study area because of the effects of topography and urbanization.

The accuracy of ET estimates from the SEBS model using MSTAT data is similar to that achieved by Gao^11^ who used the Thornthwaite water balance method to estimate the spatiotemporal characteristics of ET across the Haihe River Basin during 1960–2002.

### Spatial distribution of actual ET

**Figure 2:**
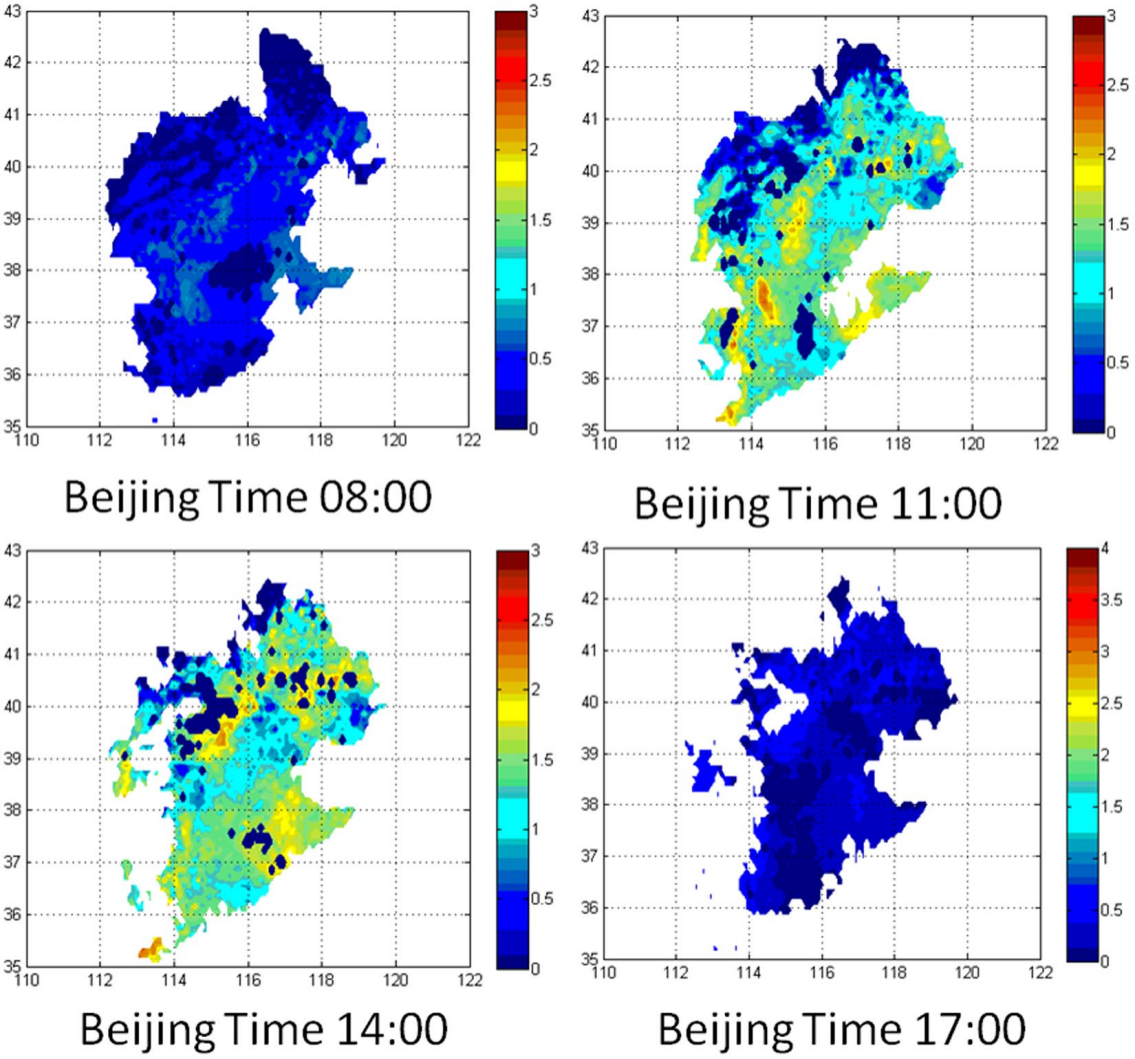
Maps of hourly ET of the Haihe River Basin on 12 September 2010 (mm/3 h) created using MATLAB. Vertical and horizontal coordinates are latitude and longitude, respectively.

**Figure 3:**
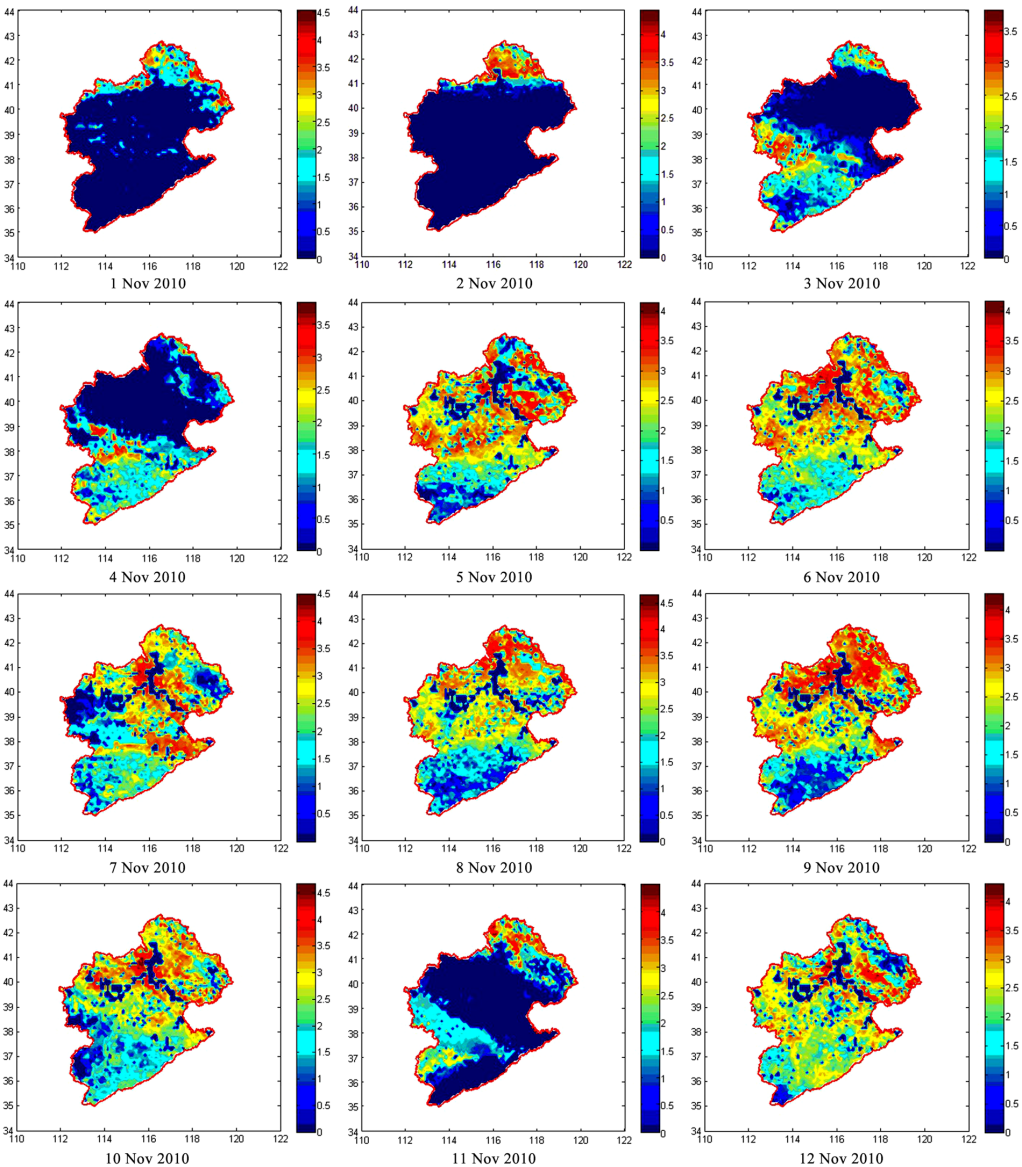
Maps of daily ET for the Haihe River Basin (mm/d) from November 1–12, 2010. Vertical and horizontal coordinates are latitude and longitude, respectively.

Several 3-hourly ET maps were constructed using the SEBS output. Daily ET maps were calculated by summing eight images of 3-hourly ET. The final hourly and daily maps of ET are shown in Figs 2 and 3, respectively. It can be seen that the largest ET is during 11:00–14:00 Beijing Time (BT) because of increased incoming solar radiation. The daily average variation of ET across the entire study area is ~1.5 mm.

Maps of daily ET can provide clear information on its spatial distribution. As can be seen from the maps of daily ET for November 1–12, 2010 (Fig. 3), ET is greatest in the east, and it decreases from east to west and from south to north across the basin. The high-value areas are mainly in the north and east near coastal regions of the study area. In northern areas, the terrain is high and there is abundant soil moisture because of extensive vegetation coverage; therefore, ET in this region is greater than in other areas.

**Figure 4:**
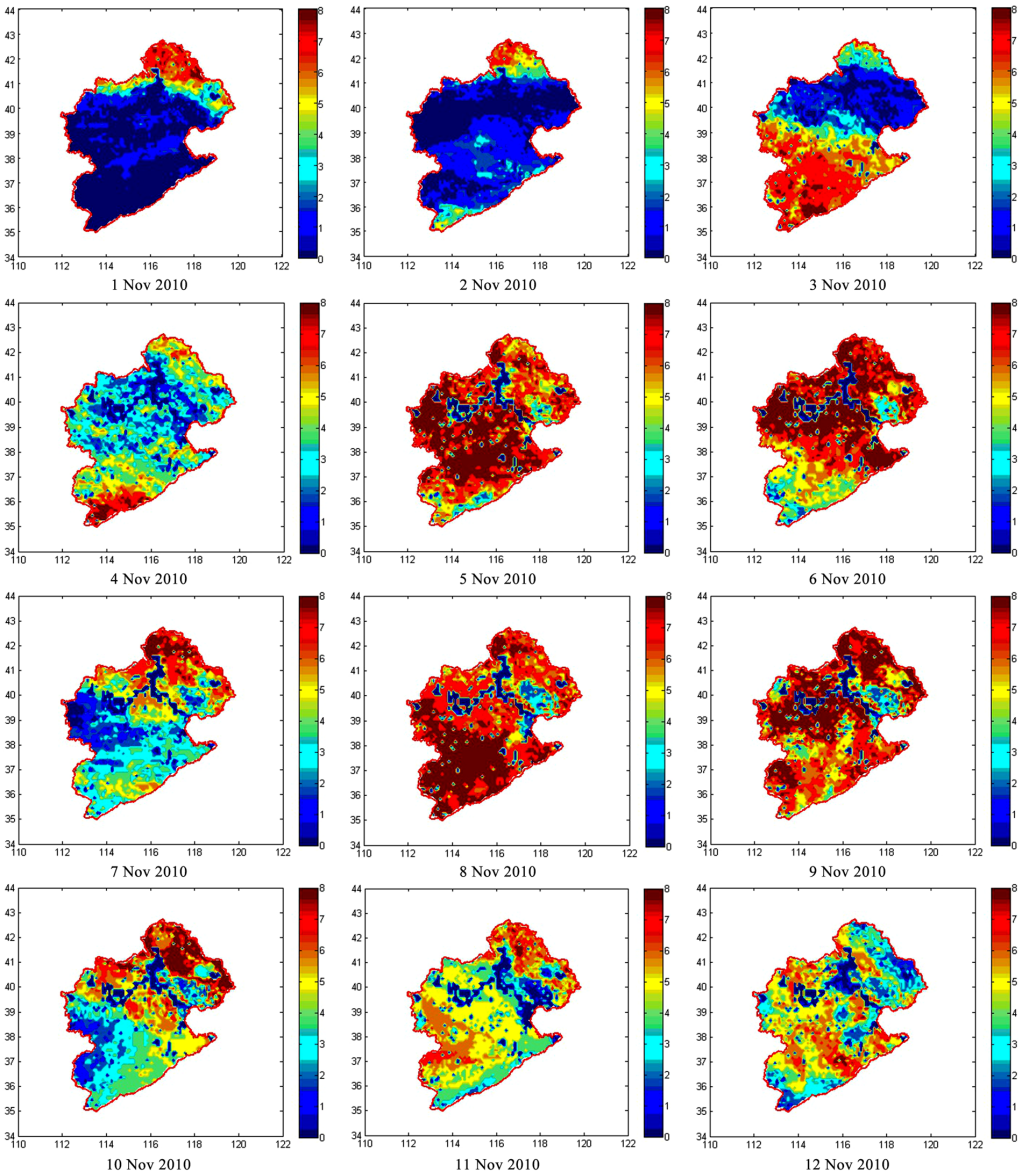
Numbers of points used to calculate daily ET from November 1–12, 2010. Vertical and horizontal coordinates are latitude and longitude, respectively.

Some areas in Fig. 3 show values of ET = N/A (e.g., November 1, 2, and 10, 2010). For the daily ET calculated by summing the eight images of 3-hourly ET, a null value on each map is assumed zero to avoid calculation errors. Therefore, areas showing small values of daily ET might indicate many null values in the 3-hourly maps. Figure 4 shows the number of effective points used to calculate daily ET. A pixel value of 8 represents a daily ET map based on eight available data. Similarly, a pixel value of 0 shows that all 3-hourly data were missing.

### ET daily variation

**Figure 5:**
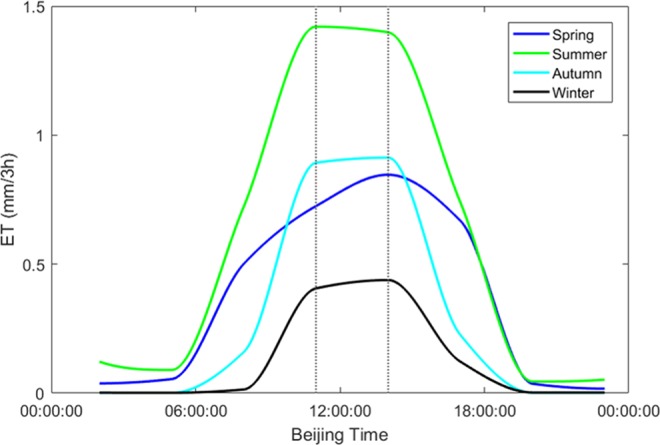
Daily variation of ET over different seasons.

The estimation of hourly ET can be used in research related to ecological environment protection and reasonable development of water resources. We analysed the daily variation of ET, which is essential in studies of climate change and water resource issues. To obtain the daily variation, we calculated the mean value of each 3-hourly period within a given day. We selected 10 days in different months to calculate the average ET representative of the four seasons: winter (1–10 January 2010), spring (1–10 April 2010), summer (10–20 July 2010), and autumn (1–10 October 2010). Figure 5 shows the daily variation of ET in the four seasons.

It can be seen from Fig. 5 that ET in all seasons is maximal around midday and minimal overnight. During summer, ET is maximal at 11:00 BT and it then gradually decreases. The daily variation of ET in summer is the largest of all seasons. The trends of ET in spring and autumn are similar, although the daily variation of ET in autumn is larger than in spring because the temperature difference between morning and night is greater. During winter, the daily variation of ET is small and much lower than in the other seasons.

## Discussion

### Spatial distribution of daily ET

The main types of vegetation cover in the Haihe River Basin are broadly croplands, grasslands, and forest. The proportion of croplands is largest, i.e., it covers approximately 51% of the total area of the study region, and this type has 6,842 pixels. The second most extensive land cover type is grasslands and wetlands (of which grasslands occupies the largest area). This type accounts for 30.79% of the total area of the study region, and it has 4,130 pixels. The third most widespread land cover type is forest, which accounts for 7.85% of the total area. Based on our understanding of the land cover types in the study area, the classification was accomplished by integrating the MOD12 data of the international geosphere-biosphere programme (IGBP) land cover classification scheme. ET over the various land cover types was analysed statistically for each day in 2010, and the detailed statistics shown in Table 2 represent the average ET of the various land cover types.

**Table 2:** Mean daily ET of different land cover types (mm/d) in 2010.

Land cover	Water	Forest	Grassland and wetlands	Croplands	Urban	Barren
ET	1.06	0.82	0.84	0.79	0.71	1.02
StDev*	0.64	0.48	0.54	0.48	0.56	0.60

*standard deviation.

As can be seen from Table 2, ET varies widely across the different land cover types in the Haihe River Basin. The maximum ET in the study area is in areas covered by water and barren land, which have strong evaporation capacity. The barren land cover type is found mainly near the coast. The strong ET associated with this type is because ET originates mainly from the ocean. Cropland, which is the most widespread land use type within the basin, has daily ET of ~0.81 mm/d. The ET of agricultural land is mainly composed of meteorological factors, soil moisture conditions, crop characteristics, and agricultural technology. Although grasslands and wetlands comprise a major land cover type in the study area, most of the area is grasslands with less vegetation coverage than croplands, and weak vegetation transpiration and serious soil and water loss produce low soil moisture. The study area covers Beijing, Tianjin, Shijiazhuang, and other counties and cities. The underlying surface of construction land is mostly impermeable and there is resistance to the evaporation of water; consequently, ET in such areas is not very high.

**Figure 6:**
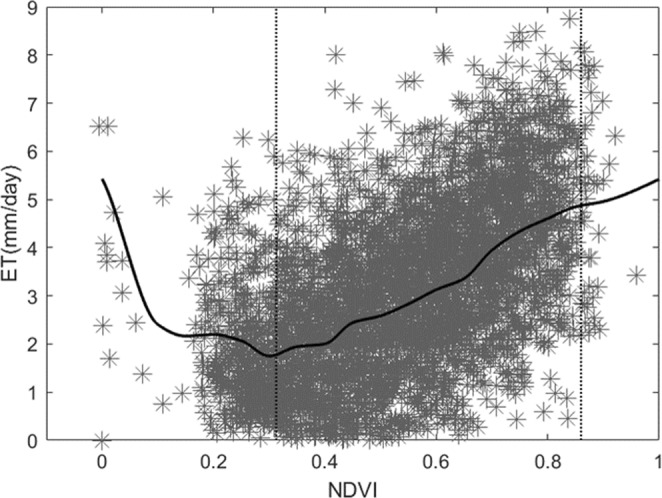
Scatterplot and curve of the relation between ET and NDVI (30 May 2013).

We also calculated the mean daily ET over the different land cover types. The results show that ET is concentrated mainly during the months of June–October. Water areas have considerable variation in ET; conversely, the variation of ET in urban areas is small. The trends of variation of ET in forests, grasslands, and croplands are similar, although the croplands cover type has a double peak because of irrigation in May. A scatterplot of MODIS-derived normalized difference vegetation index (NDVI) versus SEBS-derived daily ET (Fig. 6), and average SEBS-derived daily ET values corresponding to each 0.05 NDVI bin within 0.0–0.95 (Table 3) for May 30, 2013, are presented to discuss the relationship between ET and NDVI. It can be seen that ET values fluctuate randomly between NDVI values of 0.0 and 0.3, with the maximum ET associated with an NDVI value of 0.3. Beyond this range, ET increases linearly with further increase in the NDVI value because transpiration dominates ET. This linear increase in ET with respect to an increase in the NDVI value is found saturated beyond the NDVI value of 0.83.

**Table 3:** Average daily ET for each NDVI domain (mm/d).

Range of NDVI	Average daily ET (mm/d)	Standard deviation
0~0.05	3.74	1.57
0.05~0.10	2.4	1.89
0.10~0.15	2.16	1.68
0.15~0.20	2.19	1.15
0.20~0.25	2.06	1.29
0.25~0.30	1.74	1.18
0.30~0.35	1.94	1.26
0.35~0.40	1.99	1.22
0.40~0.45	2.44	1.46
0.45~0.50	2.58	1.36
0.50~0.55	2.83	1.43
0.55~0.60	3.12	1.43
0.60~0.65	3.35	1.49
0.65~0.70	3.94	1.42
0.70~0.75	4.33	1.58
0.75~0.80	4.6	1.48
0.80~0.85	4.84	1.57
0.85~0.90	4.95	1.62
0.90~0.95	5.15	1.52

Typically, greater NDVI values are presumed indicative of better vegetative conditions, assuming a linear relationship between the NDVI and the vegetation fraction (fc). However, the relationship might not always hold true because the NDVI is sensitive to soil background^35,36^. Hence, lower NDVI values might not be representative of vegetative conditions under an extremely open canopy or under bare soil conditions. Values of *f*_c_ of 25%–80% typically represent vegetative conditions and, for this case, we found a strong linear relationship between ET and the NDVI. Higher values of ET for low *f*_c_ or NDVI values (Fig. 6) represent water bodies that usually evaporate at a potential rate of ET. The NDVI tends to saturate under very high vegetative conditions; hence, in our case, we found that for NDVI values >0.81, the values of ET or the rate of increase in ET rate are also saturated.

### Temporal distribution of daily ET

ET is closely related to temperature and the NDVI. Our research shows that the highest daily transpiration is in summer, followed in descending order by spring, autumn, and winter. During April–June, ET is high in the study area. This occurs because there is a large area of cultivated land within the study area and agricultural irrigation enhances ET during this period. The annual distribution of actual ET in the Haihe River Basin has obvious double-peaked characteristics. The two peaks appear in May and August and a low value appears in June. The spatial distribution of ET over the Haihe River Basin found in our study is similar to that reported by Pan^37^.

The annual distribution of actual ET reflects the distribution of natural climate conditions and the field management system adopted in the Haihe River Basin during the entire year. The low land surface temperatures of the winter period of the Haihe River Basin limit evaporation capacity; thus, ET in the basin starts from November to next February. During this period, the effective soil moisture content is low, winter crops are in a dormant stage, soil of cropland cultivated by a single crop is bare and evaporation is maintained at a low level. In March, the snow begins to melt and this replenishes the soil water. The physiological activities of crops and vegetation begin to recover and ET increases significantly. The main period of crop growth and irrigation in the Haihe River Basin is during April and May. During this period, actual daily ET is reasonably high because of the sufficient soil water supply and large crop water consumption. June is the period of harvest for certain crops (e.g., winter wheat). Consequently, the croplands that account for 50% of the total area of the Haihe River Basin become bare and daily ET decreases slowly. The rainy season in the Haihe River Basin is in July and August, and the second peak of daily ET in the basin is observed during this period because of the growth of the second-season crops (e.g., summer maize) and vegetation, as well as the high summer temperatures. After this, because of changes in the hydrothermal conditions and the physiological behaviours of the vegetation, the basin-scale ET gradually decreases until November, when the return of the winter period completes the cycle.

### ET error source analysis

The study region extends along the coast and it covers a vast area with complicated topography and a variable climate. Furthermore, several cities (e.g., Beijing and Tianjin) within the basin create differences in the surface characteristic parameters and meteorological data. The elevation of the area is in the range of 0–2000 m, and the range of meteorological data increases greatly with elevation. The present research used a 0.1° forcing dataset; however, at this spatial resolution, it is difficult to accurately express the changes of terrain within a single pixel.

We used ITPCAS (Institute of Tibetan Plateau Research, Chinese Academy of Sciences) meteorological forcing data. These data were produced by merging a variety of data such as China Metrological Administration (CMA), Tropical Rainfall Measuring Mission (TRMM) satellite precipitation, Global Energy and Water cycle Experiment - Surface Radiation Budget (GEWEX-SRB), Princeton forcing, and Global Land Data Assimilation System (GLDAS) data from ground observations and reanalysis. Although this is more accurate than other forcing datasets, as indicated in the previous section, the data have some errors that influence the SEBS model output. This dataset was interpolated; however, the study area is large and such information cannot represent the characteristics of the entire atmospheric boundary layer, especially in areas with strongly undulating terrain. This causes non-zero surface temperatures when the evaporation fraction is zero.

The land surface temperature data with high temporal resolution were derived from the geostationary MTSAT. These data can only be obtained during cloud-free weather because clouds cause data gaps. In the present research, the hourly ET maps were selected from cloud-free periods. In the case of full cloud coverage, it was assumed that ET within the area was zero. Further validation of these possible errors will be performed in future work.

## Conclusions

This study estimated hourly and daily ET using the SEBS model with geostationary RS data and available meteorological observations from the Haihe River Basin. Moreover, we analysed the surface characteristic parameters from MTSAT and ITPCAS meteorological forcing datasets, both of which have the highest temporal and spatial scales among the currently available land surface research datasets. Pre-processing was performed on the two datasets to match their temporal and spatial distributions for the study area. Analysis of the land surface radiation, water and heat fluxes, and ET derived from the SEBS model was undertaken. Measurements from five flux towers located in the Haihe River Basin were collected during our fieldwork. The high quality ground observations were used to validate the model results and to analyse the errors at the flux tower points.

In summary, the 3-hourly and daily ET derived from the SEBS model performed well in the study area. The estimation of high-temporal-resolution ET using the RS method was demonstrated feasible. The model results are close to the average ET reported in related literature. This further proves that the SEBS model is both reasonable and accurate. Finally, the influences on SEBS-derived ET and possible error sources were highlighted based on the study results. In general, we can produce high-temporal-resolution land surface temperature using MTSAT satellite data.

Faced with the increasingly serious situation of water resource shortages in the Haihe River Basin, the monitoring and estimation of ET is very important for regional planning, water resource management, and sustainable development within the region. In the future, we could use improved optimization methods; however, in the meantime, it is possible to take advantage of the method proposed here that combines high-temporal-resolution and high-spatial-resolution RS data for energy balance research to improve the results.

## Methodology

### Study area

**Figure 7:**
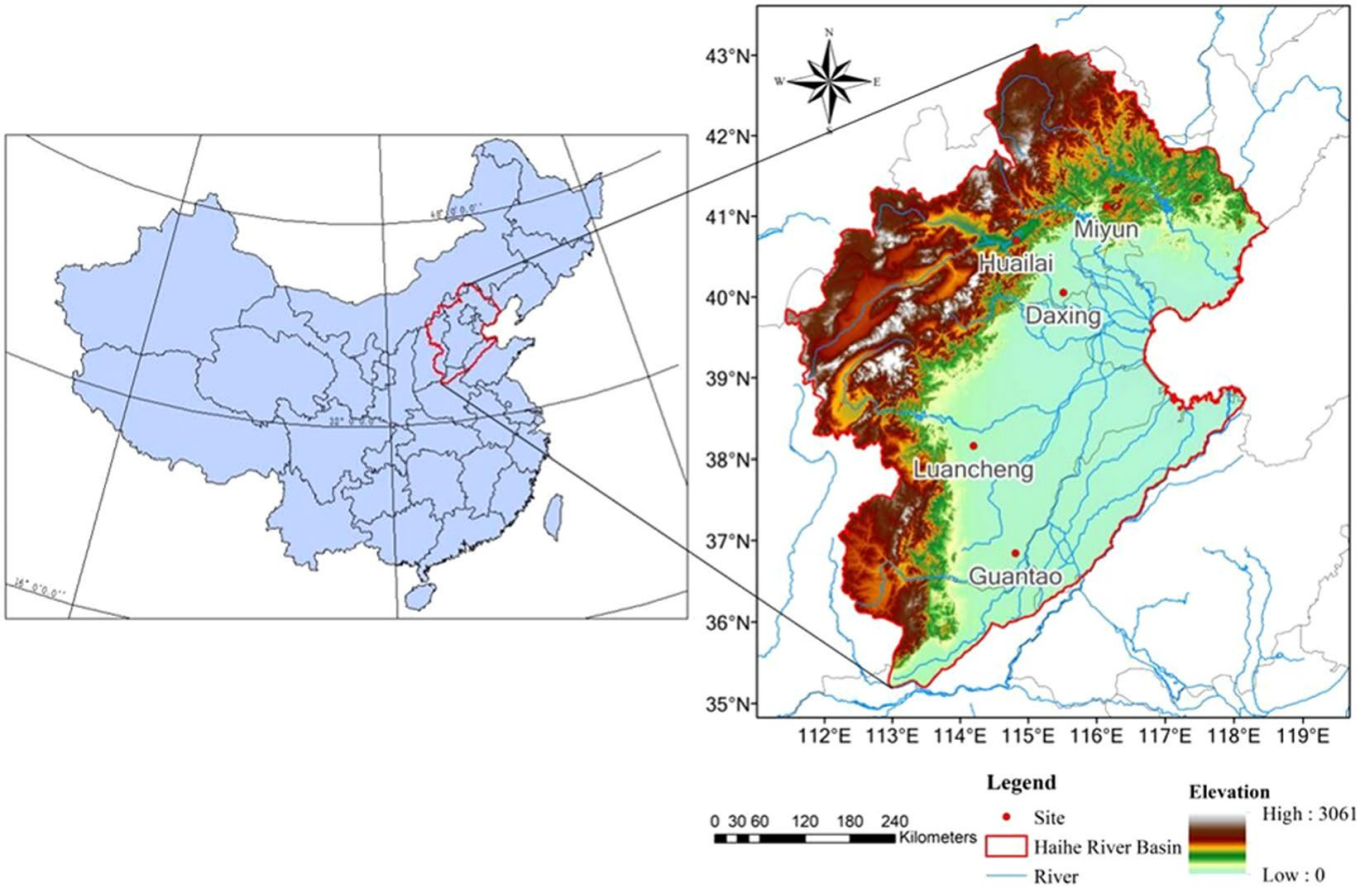
Study area of the Haihe River Basin.

The Haihe River basin is located in northern China between 35°N–41.5°N and 112°E–118.5°E (Fig. 7). The watershed covers an area of 318,200 km^2^, which accounts for 3.5% of the total area of China. The upper, middle, and lower reaches of this basin, which stretch from northern Henan Province to southern Inner Mongolia, comprise three major river systems, namely, the Luan, Haihe, and Majia. The basin covers all of the municipalities of Beijing and Tianjin, most of Hebei Province, and parts of Shandong, Henan, Shanxi, and Liaoning provinces plus the Inner Mongolia Autonomous Region^38^. Vegetation in the Haihe River Basin is mainly grasslands in Inner Mongolia, deciduous broadleaf forest in the mountainous area of North China, and warm–temperate deciduous broadleaf forest and croplands on the Haihe Plain.

The Haihe River Basin has a semi-humid and semi-arid continental monsoon climate in the warm temperate zone, which is sensitive to the effects of climate change and it is an area highly vulnerable in terms of its ecological environment. Because of the dense population of the region, the conflict between the supply and demand of water resources is prominent, and the shortage of water resources has become the main restriction on the sustainable socioeconomic development of the Haihe River Basin. ET is a key parameter of the land surface ecosystem and it is an important factor in water cycle research.

### Data collection and data validation

Fieldwork was carried out to collect the data required for the validation of ET and the land cover product, including the acquisition of flux tower data, meteorological data, and other useful data from the Haihe River Basin. The SEBS model uses a variety of satellite and meteorological forcing data to estimate energy flux. The satellite data used in our research included MTSAT and MODIS data. MTSAT is a Japanese transport satellite that succeeds the Geostationary Meteorological Satellite (GMS) series as the next generation of satellites covering East Asia and the western Pacific^39^. The images cover the earth’s surface from 5°–55°N and 75°–145°E^40^ with spatial resolution of 0.1°. This region includes most of eastern Asia. The MTSAT series provides imagery of the Northern Hemisphere at 30–min intervals. These satellites replace the GMS-5 satellite, also known as Himawari 5. MTSAT can provide imagery in five wavelength bands: visible and four infrared bands including the water vapour channel. The visible light camera has 1-km resolution and the infrared cameras have 4-km resolution (the resolution is lower away from the equator at 140°E). In comparison with GMS-5, the MTSAT series carry a new imager with a new infrared (IR4) channel (3.5–4.0 μm) in addition to four other channels (VIS, IR1, IR2, and IR3).

The China Meteorological Forcing dataset was used in our research. The new dataset was developed by the hydro-meteorological research group at the Institute of Tibetan Plateau Research, Chinese Academy of Sciences (ITPCAS)^41^. It is more accurate than other forcing datasets for calculating energy flux, according to several recent reports^42–44^. The meteorological forcing data were produced by merging a variety of data from ground-based observations and reanalysis. They contain seven variables: temperature, pressure, specific humidity, wind speed, downward shortwave radiation, downward longwave radiation, and precipitation rate. The input datasets used for calculating ET in the study area are listed in Table 4.

**Table 4:** Input datasets used for calculating ET for the Haihe River Basin.

Variables	Date source	Temporal resolution	Period
LST	MTSAT-1	1 hours	2010–2014
Air temperature		3 hours	1979–2015
Specific humidity			
Pressure			
Wind speed	China Meteorological Forcing		
Downward shortwave radiation	Dataset(ITPCAS)		
Downward longwave radiation			
Upward shortwave radiation	ITPCAS & GlobalAlbedo	3 hours	2010–2015
Upward longwave radiation	LST (MTSAT-1)	hourly	2010–2014
Canopy height	GLAS & SPOT VEGETATION	1 month	2009–2014
Albedo	GlobalAlebedo	16 days	2000–2015
NDVI	MOD13A2	16 days	2010–2014
LAI	MOD15A2	8 days	2010–2016
Land cover	MOD12Q1	yearly	2010–2014

In addition, a variety of RS and meteorological forcing data were used to estimate the energy flux. These data must be validated by comparison with independent datasets. According to research^45–48^, the energy balance measurement system (components of radiation, an eddy covariance system, and automated meteorological stations) can be used to validate the satellite image data, forcing data, and final ET product. Validation of the satellite and meteorological forcing data was done by comparing these datasets with those from five flux stations (Daxing, Miyun, Guantao, Huailai, and Luancheng) in the Haihe River Basin (see Table 5). Validation of these input data can help in the analysis and minimization of ET errors attributable to the input data.

**Table 5:** Detailed information on the flux towers.

Site Name	Location	Land cover	Maximum Vegetation Height(m)	Type	Tower Height (m)
Luancheng	7°53′21.7′′N, 114°41′34.5′′E	Maize	2.2	CPEC200	3
		Winter wheat	0.7	Li7550	
Huailai	40°20′55.9′′N, 115°47′17.5′′E	Maize	2.2	Gill & Li7500	10
		Winter wheat	0.7		
Daxing	39°37′16.7″N, 116°25′37.2″E	Maize	2.2	Li7500 and CSAT3, Li-cor and Campbell	3
		Winter wheat	0.7		
		Vegetable	0.5		
Miyun	40°37′50.82″N, 117°19′23.83″E	Orchard	4	Li7500 and CSAT3, Li-cor and Campbell	26.66
		Maize	2.2		
Guantao	36°30′54.1″N, 115°07′38.7″E	Maize	2.2	Li7500 and CSAT3, Li-cor and Campbell	15.6
		Winter wheat	0.7		
		Cotton	1.2		

**Figure 8:**
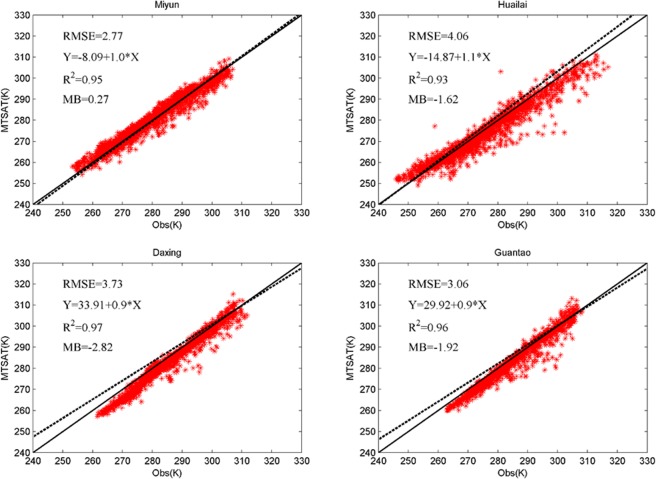
Scatter plots of LST from MTSAT against *in situ* measurements (Miyun, Huailai, Daxing, and Guantao).

The land surface temperature (LST) at each site was calculated using the Stefan–Boltzmann equation, which describes the radiation from a target (black body) in terms of its temperature. This equation is written as:

1 $${{R}_{l}}^{\uparrow }=\varepsilon \sigma T{}^{4},$$

2 $$T={({{R}_{l}}^{\uparrow }/\varepsilon \sigma )}^{\frac{1}{4}},$$

where *T* is LST, ${{R}_{l}}^{\uparrow }$ is upward longwave radiation, *ε* is “broadband” land surface emissivity (assumed as 0.975 in this study, Chen^47^), and *σ* is the Stefan–Boltzmann constant (5.67 × 10^−8^ W m^−2^ K^−4^). Comparisons of LST at four sites are shown in Fig. 8.

### Model description

The SEBS model developed by Su^22^ was used for estimation of turbulent heat fluxes and the evaporative fraction using satellite earth observation data, in combination with meteorological information at a regional scale. The SEBS model has been used widely for ET and land surface flux estimation^49–51^. We used the modified model version of Chen^48^, which revised the roughness length for heat transfer from bare ground and snow cover, making the model more suitable for application at the regional scale.

As the principle of the SEBS model, the process of energy flux exchange can be expressed as a surface energy balance equation:

3 $${R}_{n}={G}_{0}+H+\lambda E,$$

where *R*_*n*_ is net solar radiation, *G*_0_ is soil heat flux, *H* is turbulent sensible heat flux from the underlying surface to the atmosphere, *λE* is turbulent latent heat flux from the underlying surface to the atmosphere, *λ* is the latent heat of vaporization, and *E* is actual ET^22^.

The net radiation is the difference between incoming and outgoing surface shortwave and longwave radiation. The surface radiation balance equation for calculating the net radiation is

4 $${R}_{n}=(1-\alpha ){{R}_{s}}^{\downarrow }+{{R}_{l}}^{\downarrow }-{\varepsilon }_{s}\sigma {{T}_{s}}^{4},$$

where *R*_*n*_ is the net solar radiation; ${{R}_{s}}^{\downarrow }$ is downward shortwave radiation, which is the total solar radiation; ${{R}_{l}}^{\downarrow }$ is downward longwave radiation; *α* is surface albedo; *ε*_*s*_ is land surface emissivity; and *T*_*s*_ is land surface temperature. The parameters *α*, *ε*_*s*_, and *T*_*s*_ can be derived from remote sensing data from visible and thermal inferred channels, while ${{R}_{s}}^{\downarrow }$ and ${{R}_{l}}^{\downarrow }$ are obtained from meteorological observation data.

Soil heat flux is an important component that influences soil evaporation and surface energy exchange. It refers to heat exchange within the soil, determined as an unfixed percentage of energy:

5 $${G}_{0}={R}_{n}\cdot [{\varGamma }_{c}+(1-{f}_{c})\cdot ({\varGamma }_{s}-{\varGamma }_{c})],$$

where Γ_*c*_ and Γ_*s*_ are empirical coefficients under a full vegetation canopy and bare soil. An interpolation is performed between these limiting cases using the fractional canopy coverage *f*_*c*_, which can be calculated from the NDVI. The G_0_ calculation for water, snow, and urban areas can be referred to Chen^48^.

The parameter *f*_*c*_ corresponds to the fraction of ground surface covered by green vegetation canopy. For the SEBS model, *f*_*c*_ can be used to evaluate the contributions of vegetation and bare soil to the roughness parameterization. This parameter is sensitive to vegetation amount and it can be derived from the NDVI as follows:

6 $${f}_{c}=\frac{NDVI-NDV{I}_{\min }}{NDV{I}_{\max }-NDV{I}_{\min }},$$

where *NDVI*_max_ and *NDVI*_min_ represent the NDVI value for full vegetation and bare soil in a study area, respectively.

Sensible heat flux is the energy transferred to the atmosphere by turbulence because of a temperature difference between the earth’s surface and the atmosphere, and it represents some of the energy available to heat the air. To calculate sensible heat flux accurately, zero-plane displacement height *d*_0_, aerodynamic temperature, and dynamic roughness parameters must be determined. Roughness height for heat transfer is difficult to estimate because of the influence of terrain, environmental variables, and underlying surface conditions. Because of this, estimation of surface turbulent flux and the evaporation fraction has substantial uncertainty via the surface radiation temperature. The parameters *d*_0_, roughness height for momentum transfer *z*_0*m*_, and roughness height for heat transfer *z*_0*h*_ can be determined from the following equations derived by Massman^52^ and discussed in both Su^53^ and Chen^48^. In this method, *d*_0_ is modelled as follows:

7 $${d}_{0}/h=1-\frac{1}{2{n}_{ec}}[1-\exp (\,-\,2{n}_{ec})]$$

where *h* is canopy height (m), which is vital for turbulent heat simulations. This meant that deriving an accurate estimation of *h* for the Chinese landmass was very important in our study. We further developed an RS-based canopy height method^48^ to estimate the canopy height distribution across China. The parameter *n*_*ec*_ is the wind speed profile extinction coefficient in the canopy, which can be calculated as follows:

8 $${n}_{ec}=\frac{{C}_{d}\cdot LAI}{2{{u}_{\ast }}^{2}/u{(h)}^{2}}\,,$$

where *u*(*h*) is the horizontal wind speed at canopy height and *C*_*d*_ is the drag coefficient of foliage elements. *LAI* is the ratio of plant leaf area to land area per unit area, which can be derived from the NDVI. Parameter ${u}_{\ast }$ is the friction velocity and the ratio ${u}_{\ast }/u(h)$ can be derived as follows:

9 $${u}_{\ast }/u(h)={C}_{1}-{C}_{2}\exp (\,-{C}_{3}{C}_{d}\times LAI),$$

where *C*_1_ (=0.38), *C*_2_ (=*C*_*1*_ + *k/log*(0.0025)), and *C*_3_ (=15.1) are model constants related to the bulk surface drag coefficient^54^, *k* is the von Karman constant, which was considered equal to 0.4 here. Then, the values of *z*_0*m*_ and *z*_0*h*_ can be calculated by

10 $${z}_{0m}=h[1-({d}_{0}/h)]\exp (\,-\,k\cdot u(h)/{u}_{\ast }),$$

11 $${z}_{0h}={z}_{0m}/\exp (k{B}^{-1}),$$

where *B*^−1^ is the inverse Stanton number, which is a dimensionless heat transfer coefficient.

For the equation above, *kB*^−1^ is treated as a fixed value and *z*_0*h*_ is the empirical value adopted in general studies of the energy balance model. However, the SEBS model developed the parameterized method in Eq. (12) for *kB*^−1^ and it reduced the error of the scalar roughness height for heat transfer uncertainty^22^. This aspect is the main innovation of the SEBS model.

12 $$k{B}^{-1}={{f}_{c}}^{2}k{{B}_{c}}^{-1}+2{f}_{c}{f}_{s}k{{B}_{m}}^{-1}+{{f}_{s}}^{2}k{{B}_{s}}^{-1},$$

where *f*_*c*_ and *f*_*s*_ are the fractional ground surface coverages of the vegetation canopy and bare soil, respectively, and $k{{B}_{c}}^{-1}$, $k{{B}_{s}}^{-1}$, and $k{{B}_{m}}^{-1}$ are values for the canopy, bare soil, and mixed canopy and bare soil, respectively. Both $k{{B}_{c}}^{-1}$ and $k{{B}_{m}}^{-1}$ can be derived from Eq. (15), as proposed by Choudhury and Monteith^55^.

13 $$k{{B}_{c}}^{-1}=\frac{k\cdot {C}_{d}}{4\cdot {C}_{t}\cdot \frac{{u}_{\ast }}{u(h)}(1-{e}^{-{n}_{ec}/2})},$$

14 $$k{{B}_{m}}^{-1}=\frac{k\cdot \frac{{u}_{\ast }}{u(h)}\cdot \frac{{z}_{0m}}{h}}{{{C}_{t}}^{\ast }},$$

where *C*_*t*_ is the heat transfer coefficient of a leaf and ${{C}_{t}}^{\ast }$ is the coefficient of soil.

For determining the value of $k{{B}_{s}}^{-1}$, most previous research has used the method of Brutsaert^56^. However, Chen^57^ updated the calculation of $k{{B}_{s}}^{-1}$ using a superior method of roughness heat parameterization^58^. In our study, the method developed by Chen^57^ was introduced to calculate $k{{B}_{s}}^{-1}$, which has been assessed for its ability to improve the accuracy of sensible heat flux estimation:

15 $${z}_{0h}=(70\vartheta /{u}_{\ast })\exp (\,-\,7.2{u}_{\ast }^{0.5}{\theta }_{\ast }^{0.25}),$$

16 $$k{{B}_{s}}^{-1}=\,\log (\frac{{z}_{0m}}{{z}_{0h}}),$$

where *ϑ* is the kinematic viscosity of air (1.5 × 10^−5^ m^2^/s) and ${\theta }_{\ast }$ is the surface friction temperature.

Boundary layer theory has similarity relationships for profiles of the mean speed and mean temperature (${\theta }_{0}-{\theta }_{a}$). This relationship can be expressed by Eq. (17) and (19). The friction velocity, sensible heat flux, and Monin–Obukhov length (L) can be obtained by solving the following:

17 $$u=\frac{{u}_{\ast }}{k}[\mathrm{ln}(\frac{z-{d}_{0}}{{z}_{0m}})-{\psi }_{m}(\frac{z-{d}_{0}}{L})+{\psi }_{m}(\frac{{z}_{0m}}{L})],$$

18 $${\theta }_{0}-{\theta }_{a}=\frac{H}{k{u}_{\ast }\rho {C}_{p}}[\mathrm{ln}(\frac{z-{d}_{0}}{{z}_{0h}})-{\psi }_{h}(\frac{z-{d}_{0}}{L})+{\psi }_{h}(\frac{{z}_{0h}}{L})],$$

19 $$L=\frac{\rho \cdot {C}_{p}\cdot {{u}_{\ast }}^{3}\cdot {\theta }_{v}}{k\cdot g\cdot H}\,.$$

where *u* is the mean speed at reference height, *z* is height above the surface, ${u}_{\ast }$ is the friction velocity, *k* is the von Karman constant (=0.4), ψ_*m*_ and ψ_*h*_ are stability correction functions for momentum transfer and sensible heat transfer, respectively, *θ*_0_ is the potential temperature at the surface, *θ*_*a*_ is the potential temperature at height *z*, *ρ* is air density, *C*_*p*_ is specific heat capacity of air, *H* is the sensible heat flux, *L* is the Monin–Obukhov length, *g* is the acceleration of gravity, and *θ*_*v*_ is the virtual potential temperature near the surface.

After determining the sensible heat flux, the instantaneous latent heat flux and instantaneous ET from energy residual methods are available (after removal of all areas covered by clouds). Finally, an extension of the temporal scale was used to calculate hourly and daily ET according to the instantaneous ET. In addition, four tests developed by Chen^57^ were used to choose the optimal roughness length parameterization method to obtain the best ET results. Considering the overall research area, we used different methods to calculate the values of kB^−1^ of the canopy and of bare soil (see kB^−1^ calculation section). Through sensitivity and correlation analyses, we selected the optimum method to calculate the value of kB^−1^ for the study area. Further details concerning the tests are not presented in the text because of space limitations.

## Data Availability

The datasets generated during and/or analysed during the current study are available from the corresponding author on reasonable request.
